# IL-1β is not critical to chronic heart dysfunction in mice with Chagas disease

**DOI:** 10.3389/fimmu.2022.1010257

**Published:** 2022-10-14

**Authors:** Camila Victória Sousa Oliveira, Oscar Moreno-Loaiza, Daniel Figueiredo-Vanzan, Isalira Peroba Ramos, Hilton Mata-Santos, Marcelo Torres Bozza, Claudia Neto Paiva, Emiliano Medei

**Affiliations:** ^1^ Departamento de Imunologia, Instituto de Microbiologia Paulo de Góes, Universidade Federal do Rio de Janeiro (UFRJ), Rio de Janeiro, Brazil; ^2^ Institute of Biophysics Carlos Chagas Filho, Federal University of Rio de Janeiro (UFRJ), Rio de Janeiro, Brazil; ^3^ Faculdade de Farmácia, Universidade Federal do Rio de Janeiro (UFRJ), Rio de Janeiro, Brazil; ^4^ National Center for Structural Biology and Bioimage (CENABIO), Federal University of Rio de Janeiro (UFRJ), Rio de Janeiro, Brazil; ^5^ D’Or Institute for Research and Education (IDOR), Rio de Janeiro, Brazil

**Keywords:** Chronic Chagas cardiomyopathy, interleukin-1beta, mice, Trypanosoma cruzi, cardiomyopathy, heart

## Abstract

Long after *Trypanosoma cruzi* infection, 40% of individuals develop a progressive chronic chagasic cardiomyopathy (CCC), with systolic dysfunction and arrhythmias. Since we previously showed IL-1β mediates the development of systolic dysfunction and cardiac arrhythmias in diabetes mellitus and cardiorenal syndrome, and IL-1β remains elevated in Chagas disease patients, here we tested the role of IL-1β in CCC using a mouse model. Mice deficient in IL-1R expression (*Il-1r^−/−^*) survived acute *T. cruzi* infection with greater parasitemia than controls but did not lose weight as wild-type (WT) did. At the chronic stage, WT presented prolonged ventricular repolarization intervals (QJ), while *Il-1r^−/−^* presented intervals like noninfected controls. Infected *Il-1r^−/−^* and WT did not differ in stroke volume (SV), the incidence of cardiac arrhythmias on electrocardiography (EKG), whole heart action potential duration (APD), or the incidence of triggered activity after S1–S2 protocol, which is a measure of susceptibility to cardiac arrhythmias. We also treated chronically infected WT mice with an IL-1R antagonist, anakinra. Treatment shortened the QJ interval but did not improve the SV or the incidence of cardiac arrhythmias on EKG. Anakinra failed to reduce triggered activity following the electrical extra-stimulation protocol. In conclusion, the absence of functional IL-1β/IL-1R signaling did not prevent or reverse the decrease of SV or the incidence of cardiac arrhythmias induced by chronic *T. cruzi* infection, implying this is not a critical mechanism in generating or maintaining CCC. Since similar cardiac abnormalities were previously credited to IL-1β signaling, ruling out this mechanism is important to discourage further attempts of IL-1β blockade as a therapeutical measure.

## Introduction

*Trypanosoma cruzi* molecules are recognized by several pattern recognition receptors (PRRs) such as Toll- (TLRs), C-type lectin- (CLRs), and NOD-like receptors (NLRs), triggering an inflammatory response from innate immunity cells right after contamination. The inflammatory response during the acute phase contributes to activating innate trypanocidal mechanisms and prime/polarizing the T-cell response. After the establishment of an adaptative response, parasite burden is controlled, but not eliminated, and inflammation persists in several tissues such as the heart throughout the life of the chronically infected animal. In humans and in mice, myocarditis might progress after infection, producing heart disease. Systolic dysfunction, bradycardia, atrial fibrillation, ventricular tachycardia, and sinoatrial and atrioventricular blocks are all common findings in those individuals affected by chronic chagasic cardiomyopathy (CCC) (1, 2).

The inflammasome is a sensor of both microbe infections and sterile damage, which produces several function adaptations in the cardiovascular system (3, 4). Systolic dysfunction may arise from inflammasome activation and IL-1β production (5–8). In fact, several randomized clinical trials have addressed the potential of IL-1β blockade to treat heart failure (9–13). Also, arrhythmias may result from inflammasome activation, such as in atrial fibrillation driven by NOD-, LRR-, and pyrin domain-containing protein 3 (NLRP3) inflammasome activation in post–open-heart surgery (14) and in obesity (15). We have previously shown that renal ischemia-reperfusion triggers cardiac arrhythmia through NLRP3 inflammasome activation and IL-1β production (16), and in diabetes mellitus, the NLRP3 inflammasome connects metabolic dysfunction to cardiac arrhythmias through IL-1β production (17). Situations in which IL-1β works as a soluble intermediary linking inflammasome activation and cardiac dysfunction are particularly suitable for therapeutic intervention using a soluble antagonist of IL-1R, anakinra (Kineret).

The inflammasome is activated during *T. cruzi* infection. Despite surviving acute infection with Y strain *T. cruzi*, mice deficient in NLRP3 and caspase-1 present greater parasitemia than wild-type controls (18). Accordingly, macrophages from *Nlrp3* ***^−/−^*** and *Caspase-1* ***^−/−^*** mice infected *in vitro* with *T. cruzi* are less efficient in controlling parasite burden because of decreased autophagy and nitric oxide (NO) production (18, 19). The levels of IL-1β are decreased in plasma from infected *Nlrp3* ***^−/−^*** and *Caspase-1* ***^−/−^*** mice, and in fact, while NLRP3, caspase-1, and IL-18 contribute to NO production and parasitism control, IL-1β activity has no effect on macrophage parasitism (18). In mice infected with the Tulahuen strain, clearance of liver parasites is dependent on NLRP3 expression (20). Hepatic macrophages from infected *Nlrp3* ***^−/−^*** produce more reactive oxygen species (ROS) than that from wild-type mice (20), a factor that might underlie this susceptibility since oxidative stress fuels *T. cruzi* infection (21, 22). The secretion of IL-1β persists during chronic *T. cruzi* infection in patients with heart disease (23), but its role in CCC remains unknown.

Here, we tested whether IL-1β contributes to systolic dysfunction and cardiac arrhythmias in CCC. For this purpose, we infected *Il-1r* ***^−/−^*** and WT mice, and in addition, we treated chronically infected WT mice with the IL-1R-antagonist anakinra, assessing their cardiac function by electrocardiography (EKG) and echocardiography (ECHO). We also studied ventricular action potential at the whole heart level and the susceptibility to triggered activity after electrical stimulation protocol. Our results indicate that IL-1β is not critical to the generation/maintenance of cardiac arrhythmias or systolic dysfunction found in CCC.

## Results

### The absence of a functional IL-1R leads to increased parasitemia but allows weight gain in Chagas disease

To determine whether IL-1β was involved in the genesis of chronic heart dysfunction in mice, we infected young *Il-1r* ***^−/−^*** and wild-type (WT) mice (24) (6–8 weeks old, C57BL/6 background) with the Colombian strain of *T. cruzi* and monitored parasitemia and weight gain during the acute and chronic stages of infection.

**Figure 1:**
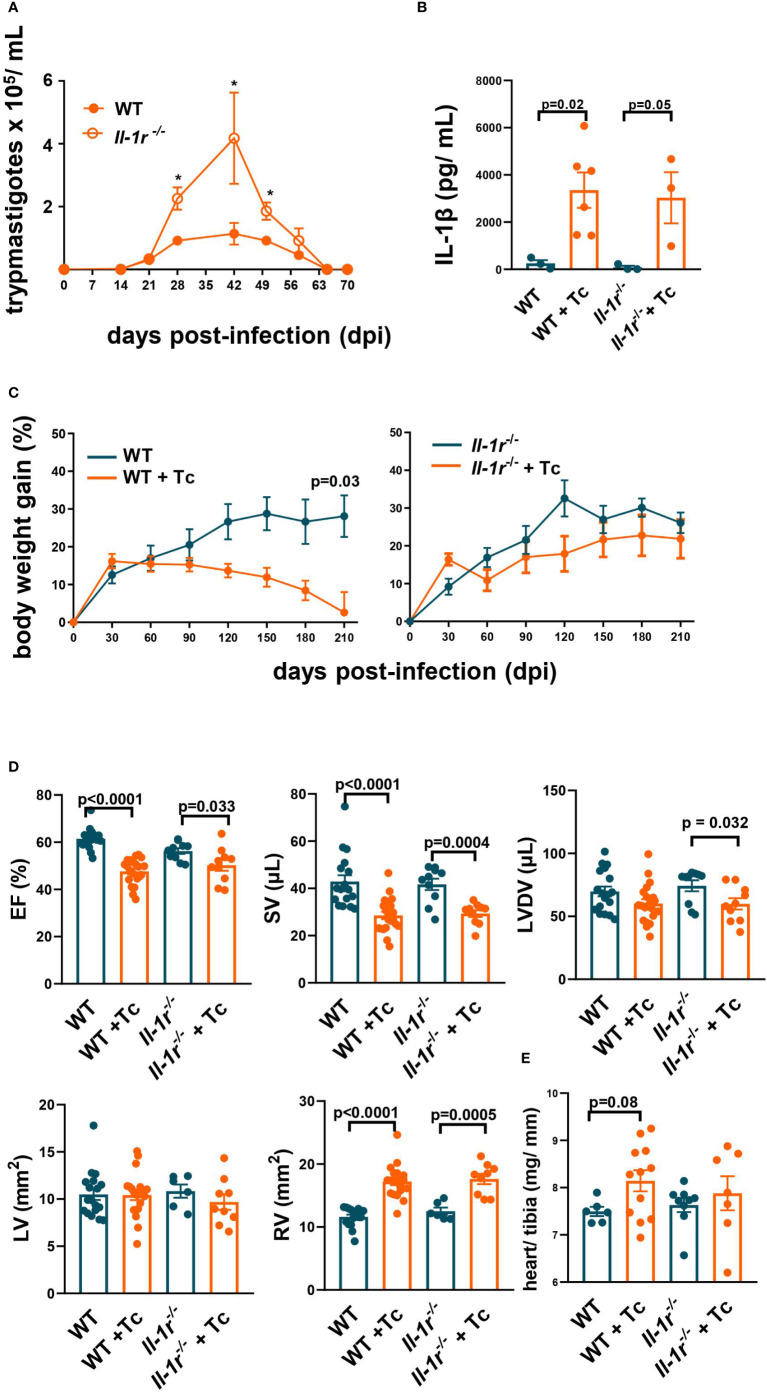
IL-1β signaling through IL-1R is active during the acute stage, and its constitutive absence is not critical to cardiac function in the chronic stage. Wild-type (WT) and *Il-1r* ^−/−^ mice (C57BL/6 background) were infected with the Colombian strain of *T. cruzi* (sum of two independent experiments with similar parasitemias, 50 and 200 parasites/inoculum) at 6–8 weeks of age. **(A)** Parasitemia (4–10 mice per group). **(B)** Serum levels of IL-1β (210 dpi, 3–6 mice per group). **(C)** Body weight gain (3–10 mice per group). **(D)** Echocardiography (210–270 dpi, 10–17 mice per group): ejection fraction (EF), stroke volume (SV), left ventricle diastolic volume (LVDV), right ventricle area (RV), and left ventricle area (LV). **(E)** The relative weight of the heart (heart weight mg/tibia length mm). Data represent the mean ± SEM. ^*^ *p* < 0.05.

During the acute stage (60 days postinfection (dpi)) *Il-1r* ***^−/−^*** mice reached greater parasitemia than WT mice (**Figure 1A**), confirming that IL-1β signaling during the acute stage contributes to reducing parasite burden. Total mortality at 210 dpi did not differ between groups (1/11 for infected *Il-1r* ***^−/−^*** mice and 1/19 for WT (*p* = 0.68)).

The plasma levels of IL-1β were significantly increased in *T. cruzi*-infected WT and *Il-1r* ***^−/−^*** mice compared to respective noninfected controls at 210 dpi (**Figure 1B**), demonstrating that the secretion of this cytokine is stimulated and remains elevated long after infection onset.

Infected WT mice started to lose weight a few months after infection and at 210 dpi had no increase in weight compared to the basal 6–8 weeks level. In contrast, noninfected WT controls continued to gain weight, as did both infected and noninfected *Il-1r* ***^−/−^*** mice (**Figure 1C**).

Taken together, these results indicate that IL-1β signaling through IL-1R contributes to controlling parasite growth and to the previously described cachexia during acute Chagas disease (25).

### The absence of a functional IL-1R prevents both bradycardia and prolongation of ventricle repolarization interval but is not involved in other Chagas disease arrhythmias

The typical findings at the chronic stage of murine Chagas disease are a decrease in ejection fraction (EF), stroke volume (SV), dilation of the right ventricle (RV), bradycardia, and an increase in the duration of P wave, PR interval, and QJ interval. These alterations mimic the clinical stages B1–B2 described in humans (26). We assessed the cardiac function of infected WT and *Il-1r* ***^−/−^*** by EKG and ECHO during chronic Chagas disease (210–270 dpi, pooled data). We compared noninfected versus infected mice among both WT and *Il-1r* ***^−/−^*** groups and also infected WT versus infected *Il-1r* ***^−/−^*** mice.

The alterations in ventricular function previously described by us at the chronic stage of the Colombian infection in BALB/c (27) were found here in the C57BL/6 background (WT, by 210–270 dpi): a significant decrease in EF and SV, accompanied by RV dilation (**Figure 1D**). Similar alterations were also found in infected *Il-1r* ***^−/−^*** mice (**Figure 1D**). A slight (9%), nonsignificant (*p* = 0.08) increase in the relative weight of the heart (heart mg/tibia mm) was observed in WT infected versus noninfected, but less so in *Il-1r* ***^−/−^*** infected versus noninfected mice (**Figure 1E**).

**Figure 2:**
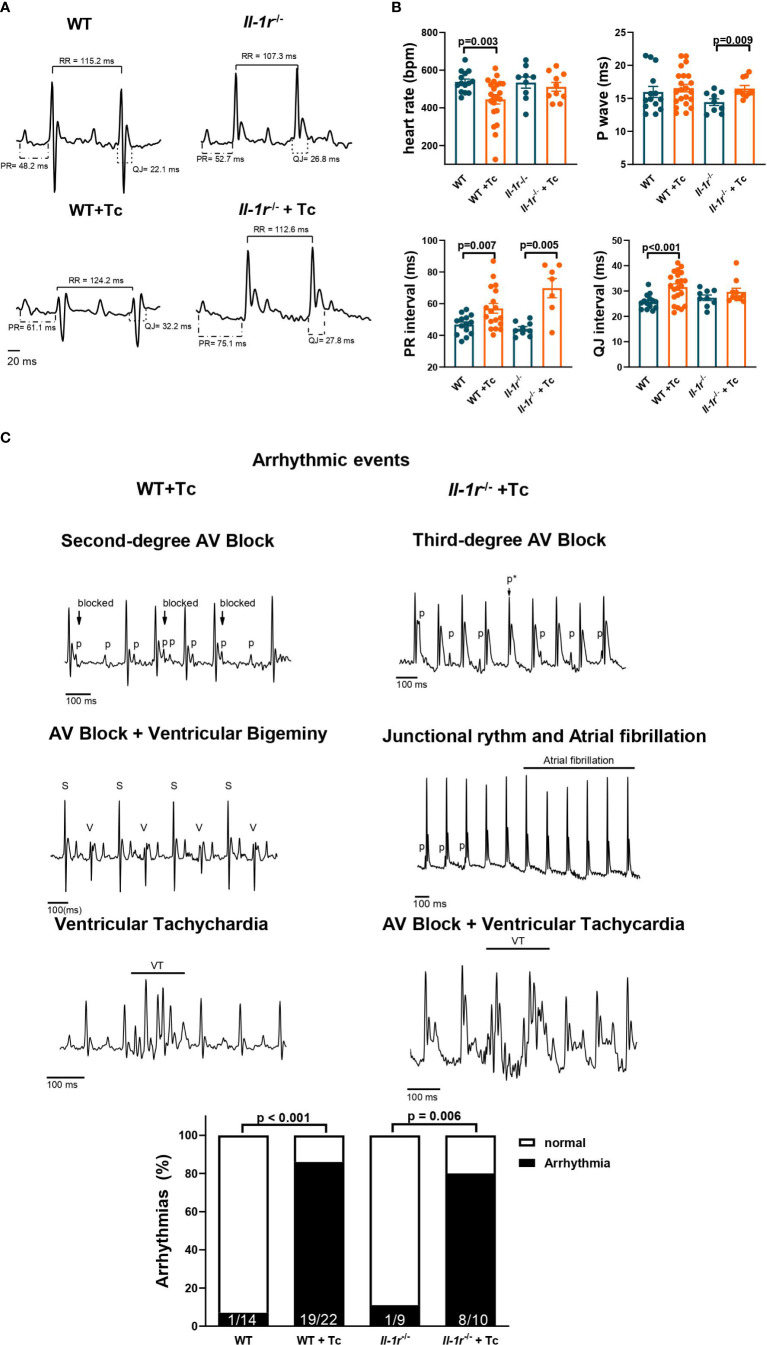
IL-1R deficiency prevents bradycardia and prolongation of ventricle repolarization but is not involved in Chagas disease arrhythmias. Wild-type (WT) and Il-1r−/− mice (C57BL/6 background) were infected with the Colombian strain of T. cruzi (sum of two independent experiments with similar parasitemias, 50 and 200 parasites/inoculum). Electrocardiography was performed at the chronic stage (210–270 dpi). **(A)** Representative EKG tracings; **(B)** heart rate, P wave, PR interval, and QJ interval duration (9–22 mice per group, data represent mean ± SEM). **(C)** Identified arrhythmic events and incidence of arrhythmias among chronically infected mice. Atrioventricular block (AV block), sinus complex (S), ventricular complex (V), ventricular tachychardia (VT), p wave (p), masked p wave (p*).

At the chronic stage, while infected WT mice presented bradycardia, infected *Il-1r* ***^−/−^*** mice preserved a heart rate like noninfected controls (**Figure 2A**). The duration of the P wave was significantly increased in infected *Il-1r* ***^−/−^*** mice compared to noninfected controls, but not in infected WT mice (**Figure 2B**). In addition, the PR interval was 58% greater in infected *Il-1r* ***^−/−^*** compared to noninfected mice, but only 22% in infected WT mice, indicating that the absence of a functional IL-1R causes a trend toward decreased conduction speed from the SA node to the Bundle of His in Chagas disease. On the other hand, the QJ interval (early repolarization, which is produced by K^+^ efflux and once prolonged, sensitizes to ventricular arrhythmias (28)) was significantly prolonged in infected WT but not in infected *Il-1r* ***^−/−^*** mice, indicating that IL-1β signaling through IL-1R is critical to prolonging ventricle repolarization interval in Chagas disease (**Figures 2A, B**).

The EKG traces of infected and noninfected mice are shown in **Figure 2A**, with intervals depicted. A low-voltage QRS was previously described in both human and murine Chagas disease and found in both infected WT and *Il-1r* ***^−/−^*** traces (**Figure 2A**).

Several arrhythmic events were detected in infected mice, such as second- and third-degree atrioventricular (AV) block, ventricular and supraventricular extrasystole, ventricular tachycardia, and traces with combined arrhythmias, such as AV block + ventricular tachycardia and AV block + ventricular bigeminy (**Figure 2B**). An even distribution of different arrhythmic events was found among infected WT and *Il-1r* ***^−/−^*** mice (**Supplementary Table SI** depicts each kind of arrhythmic event detected by us, its prevalence among infected mice in %, and the size of the mouse group *n*). The percentage of mice presenting arrhythmias was similar between infected WT and *Il-1r* ***^−/−^*** mice (**Figure 2C**).

These results indicate that IL-1β signaling through IL-1R produces adaptations in heart rate and ventricle repolarization in Chagas disease but is not directly involved in producing other arrhythmias.

### Action potential duration and ventricular-triggered activities do not depend on IL-1R in Chagas disease

As described by Medei et al. (29, 30), CCC produces alterations in ventricular repolarization. We have also previously demonstrated that IL-1β represents the immunological link between ventricular arrhythmias and type-1 diabetes. Here, we tested whether IL-1β is involved in the ventricular arrhythmias described in CCC.

**Figure 3:**
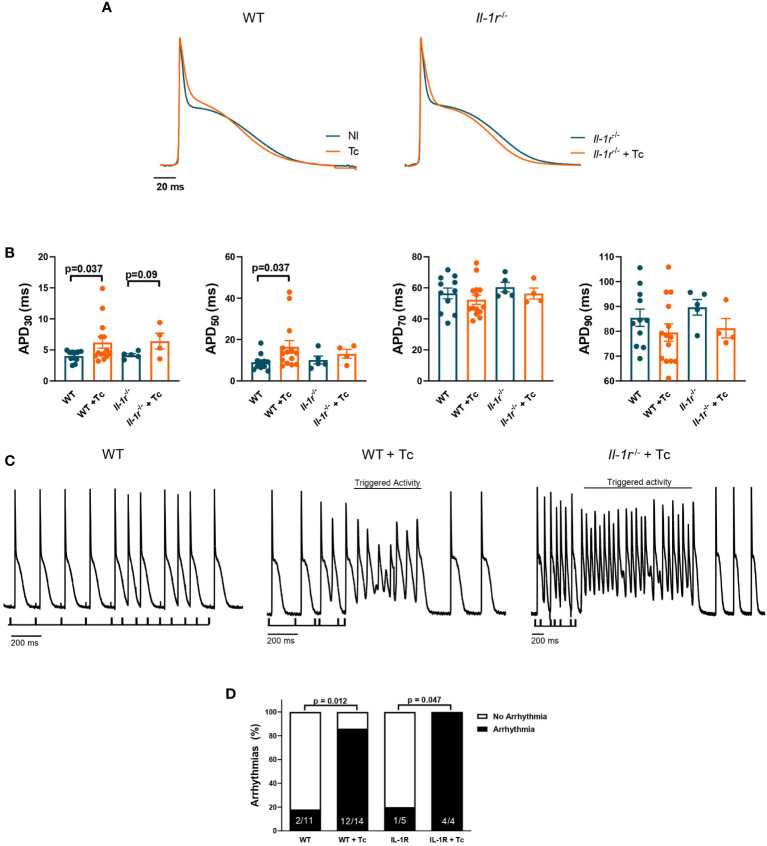
IL-1R deficiency produces slight differences in APD but not enough to prevent the susceptibility to ventricular arrhythmias in CCC. Wild-type (WT) and *Il-1r* ^−/−^ mice (C57BL/6 background) were infected with the Colombian strain of *T. cruzi* (sum of two independent experiments with similar parasitemias, 50 and 200 parasites/inoculum) and analyzed at 270 dpi. Cardiac action potential (AP) was evaluated from the endocardial layer of the left ventricle *ex vivo* heart to compare noninfected and infected mice. **(A)** Representative whole heart AP traces. **(B)** AP duration at 30%, 50%, 70%, and 90% of repolarization (4–14 mice per group). **(C)** Ventricular arrhythmic events induced by the S_1_–S_2_ protocol (4–14 mice per group, data represent mean ± SEM). **(D)** The prevalence of ventricular arrhythmic events in whole hearts from groups of mice.

An increase in ventricular action potential duration (APD) (210 dpi) at 30% (APD 30, *p* = 0.037) and 50% (APD 50, *p* = 0.037) of repolarization was observed comparing WT infected versus noninfected hearts (**Figures 3A, B**). These data indicate that phase 1 of AP is slower in infected than in noninfected WT mice (**Figure 3A**). A similar, though less pronounced phenomenon occurred among infected versus noninfected *Il-1r* ***^−/−^*** hearts at APD 30 (*p* = 0.09). Nevertheless, APD 70 and APD 90 presented a nonsignificant trend towards decreased mean values in infected versus noninfected hearts from both the WT and *Il-1r* ***^−/−^*** groups.

We tested the susceptibility to ventricular arrhythmic events, like triggered activities and early- or delayed-afterdepolarizations, using an electrical extra-stimulation protocol (S_1_–S_2_), which gradually shortens the interval between S_1_ and S_2_ stimuli, paced at 5 Hz. Hearts from infected WT and *Il-1r* ***^−/−^*** mice presented similar susceptibility to triggered activity (**Figure 3C**). Also, the S_1_–S_2_ protocol evoked a close incidence of ventricular arrhythmias in whole hearts from the WT and *Il-1r* ***^−/−^*** groups (**Figure 3D**), 85.7% of the hearts from WT+Tc were affected, while among *Il-1r* ***^−/−^*** +Tc mice, 100%.

These results demonstrate that while the lack of IL-1R might produce slight differences in APD, it is not enough to prevent ventricular susceptibility to arrhythmias, suggesting that other players are more critically involved in this process.

### Treatment with anakinra does not interfere much with ventricular function

Treatment with anakinra prevented new heart failure events in experimental and randomized clinical trials (31). Since the outcome of infection in the constitutive absence of IL-1R reflect the role of IL-1β in the generation of CCC, which based on our results, seems to be of little relevance, we performed IL-1R blockade in infected WT mice. Targeting IL-1R with anakinra offers a translational opportunity to test whether it can reverse established CCC and thus have a therapeutical potential. We treated C57BL/6 mice with well-established CCC (240-270 dpi, pooled data) with anakinra, for 30 days, and assessed their ventricular function.

**Figure 4:**
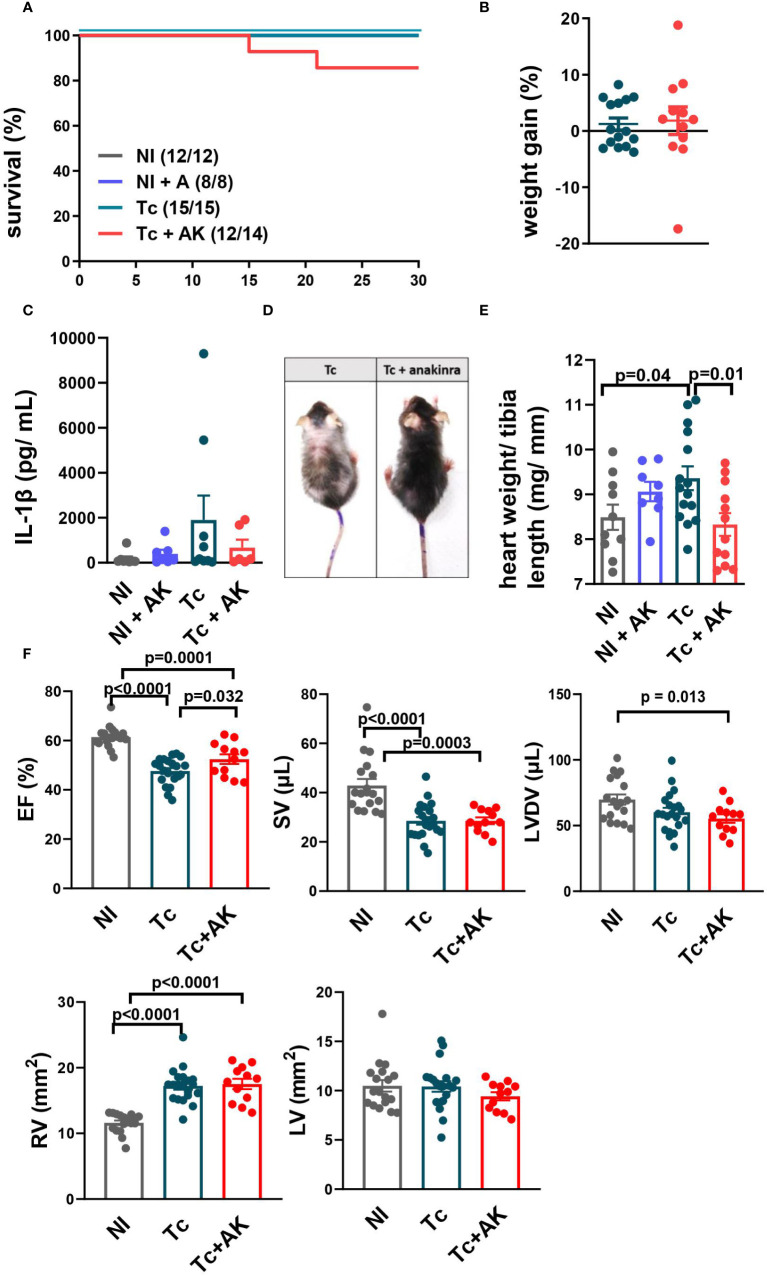
Treatment of chronically infected mice with anakinra (IL-1R antagonist) improves general health but not systolic function. C57BL/6 mice were infected with the Colombian strain of *T. cruzi* (100–200 parasites) and at chronic stage (240–270 dpi), treated with anakinra (10–25 mg/kg, i.p., sum of two independent experiments with 100 and 200 parasites/inoculum). **(A)** Survival curve. **(B)** Percentage of body weight gain. **(C)** Serum levels of IL-1β. **(D)** Less pronounced disease signals such as prostration, bald spots, ruffled fur and hunched back. **(E)** The relative weight of the heart (heart mg/tibia mm). **(F)** Echocardiography: ejection fraction (EF), stroke volume (SV), left ventricle diastolic volume (LVDV), right ventricle area (RV), and left ventricle area (LV). Data represent the mean ± SEM from 11 to 15 mice per group. NI, noninfected; Tc, chronically infected mice; Tc+AK, chronically infected mice treated with anakinra.

Though treatment with anakinra did not significantly alter survival (**Figure 4A**), weight gain (**Figure 4B**), or IL-1β plasma levels (**Figure 4C**) during the chronic stage, there was a visual clinical improvement, with less pronounced disease signals such as prostration, bald spots, ruffled fur, and hunched back (**Figure 4D**). Such general health improvement was accompanied by a normal relative heart weight, contrasting with increased ones found among nontreated infected controls (**Figure 4E**).

Treatment of infected mice with anakinra recovered EF (**Figure 4F**), but this recovery was neither due to a mean increase in SV nor to a mean decrease in LVDV, which did not differ significantly from nontreated infected controls but produced increased SV/LVDV ratios (EF).

A similar RV dilation was found in both infected groups, nontreated and anakinra-treated mice (**Figure 4F**). No differences were found in LV among groups.

Taken together, these results show anakinra did not significantly improve cardiac ventricular function, even though general health seemed to have improved.

### Treatment with anakinra does not greatly alter ventricular arrhythmia susceptibility or cardiac conduction disturbances

Treatment with anakinra reverses cardiac electrical disturbances in T1 diabetes mellitus (17) and cardiorenal syndrome (16) in mice. Since in Chagas disease the IL-1β levels are increased (23), as we also observed here, we tested the hypothesis that anakinra would reverse the electrical disturbances induced by *T. cruzi* infection in a well-established CCC model.

**Figure 5:**
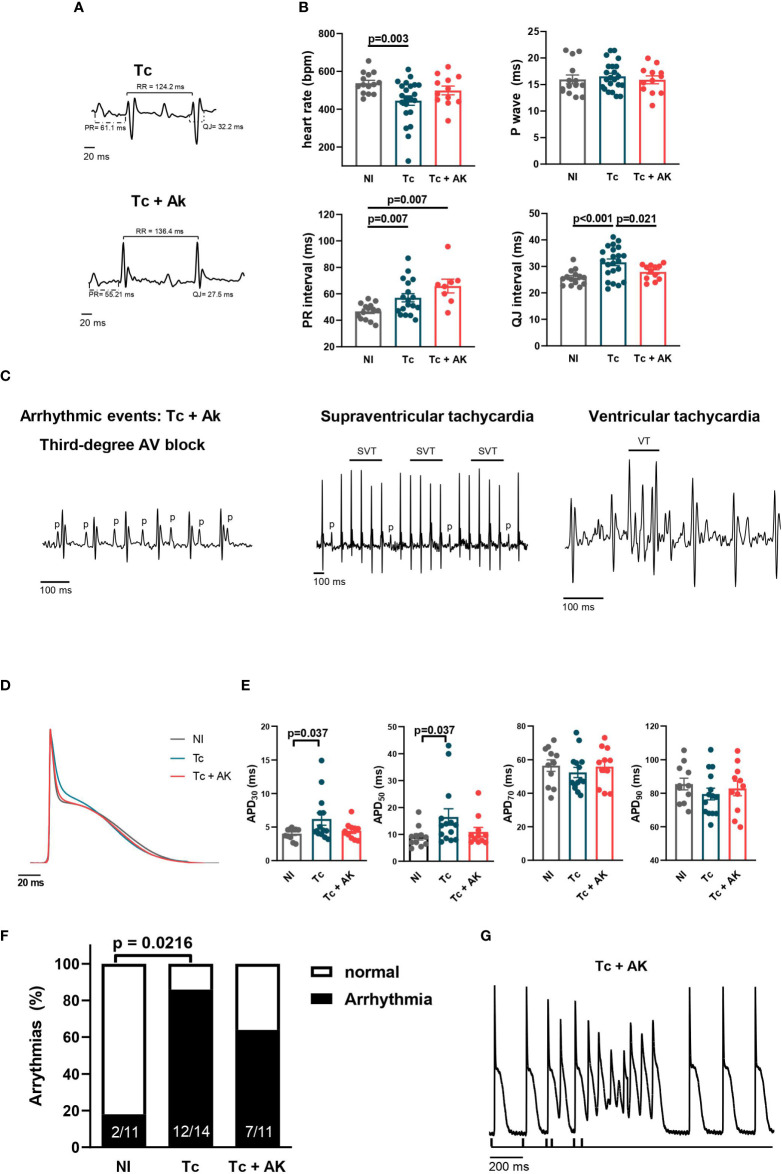
Treatment of chronically infected mice with anakinra (IL-1R antagonist) does not reverse arrhythmias. Wild-type (WT) (C57BL/6 background) were infected with the Colombian strain of *T. cruzi* (100 parasites) at 270 dpi and treated with an IL-1R antagonist, anakinra (10 mg/kg, i.p.), daily for 30 days. **(A)** Representative EKG tracings; **(B)** heart rate, P wave, PR interval, and QJ interval duration (9–22 mice per group, data represent the mean ± SEM). **(C)** Identified arrhythmic events. **(D)** Representative whole heart AP traces; **(E)** whole heart AP duration at 30%, 50%, 70%, and 90% of repolarization (4–14 mice per group). **(F)** Incidence of triggering activity among hearts from groups of mice upon S_1_–S_2_ protocol; **(G)** representative tracing showing triggering activity in a Tc+AK heart. NI, noninfected; Tc, chronically infected mice; Tc+AK, chronically infected mice treated with anakinra.

Treatment of *T. cruzi*-infected mice with anakinra for 30 days starting at the chronic stage (240–270 dpi, pooled data) shortened ventricular repolarization interval (QJ, **Figures 5A, B**), an important electrical disturbance described in CCC, and reduced diffuse and focal inflammatory infiltrates/associated interstitial and perivascular fibrosis (**Supplementary Figure 1**). However, a prolonged PR interval was still observed in hearts from anakinra-treated mice, like those in infected, untreated mice. Moreover, infected mice treated with anakinra presented a similar percentage of ventricular arrhythmic events and similar abnormal electrical conduction when compared to infected untreated mice (**Figure 5C**).

To study in depth the cellular mechanisms involved in shortening QJ interval, an AP recording in the left ventricle was performed in isolated whole hearts. Treatment with anakinra was able to reverse the changes induced in the APD at the chronic stage (**Figures 5D, E**). However, at an intact heart level, the susceptibility to triggered activities upon extra-stimulation protocol (S_1_–S_2_) was not significantly reduced in the left ventricle of mice treated with anakinra (63.6%) when compared to infected, untreated mice (85.7%) (**Figures 5F, G**).

Collectively, our results indicate that IL-1β signaling through IL-1R is involved in delayed ventricular repolarization but is not critical to produce ventricular arrhythmic events.

## Discussion

Here, we assessed the role of IL-1β in ventricular and electrical function during the chronic stage of Chagas disease. We found a shortening of the ventricular repolarization interval (QJ) in infected *Il-1r* ***^−/−^*** compared to WT mice and also in infected WT after treatment with anakinra. However, there was no reduction in the incidence of arrhythmias in chronically infected *Il-1r* ***^−/−^*** mice or after treatment of chronically infected WT mice with anakinra. The triggered activities upon extra-stimulation protocol (S_1_–S_2_) did not change in response to *in vivo* blockade of IL-1β. Blockade of IL-1β signaling did not improve stroke volume. These results indicate that IL-1β is not critically involved in the genesis and maintenance of Chagas disease cardiac dysfunction.

The role of IL-1β production in adapting heart function to both sterile and infectious inflammatory conditions has been extensively described in the literature (7, 32, 33). An ongoing phase 2 clinical trial is even assessing the therapeutical potential of IL-1R blockade with anakinra to treat heart failure (34). Along with TNF and IL-6, IL-1 (not mature IL-1β) was indicated as a factor involved in delayed repolarization, long QT syndrome, and ventricular tachycardia (35). In 2016, we demonstrated that exposure of isolated ventricular rat cardiomyocytes to IL-1β reduced transient outward potassium current (I_to_), a key repolarizing current, thereby prolonging APD and providing a substrate for triggered arrhythmogenic activity (17). Exposure to IL-1β increased diastolic leak of Ca^+2^ from the sarcoplasmic reticulum (SR) and spontaneous contractile events, an index of arrhythmic events. Also, the absence of a functional IL-1R expression prevents QT prolongation, and treatment with anakinra decreases arrhythmia scores in diabetes mellitus. In renal ischemia/reperfusion, IL-1β produced after sensing kidney injury prolongs QJ and sensitizes to cardiac arrhythmias (16). Therefore, there is a strong case for IL-1β being involved in the genesis and maintenance of heart insufficiency and cardiac arrhythmias, but we found here that it is not the case for CCC.

Since other studies found a role for TNF in ventricular and electrical function in CCC (36), and since TNF, IL-1β, and IL-6 are often involved in ventricular tachycardia (37–39), we believe the focus of future studies should be to simultaneously target TNF, IL-6, and IL-1β (40) in order to assess the role of these usually cooperative cytokines in CCC.

While infection of *Il-1r* ***^−/−^*** mice provides a model in which no IL-1β signaling is allowed since the onset of infection, it fails to exclude differences in heart physiology/immunological response that might reflect the role of IL-1β in mouse development. However, both infection of *Il-1r* ***^−/−^*** mice and IL-1R blockade with anakinra starting at chronic infection resulted in reduced QJ compared respectively to WT or nontreated controls. These data indicate that IL-1β production evoked by infection is involved in generating and maintaining a prolonged QJ interval in CCC.

Several health parameters pointed towards a general improvement in the absence of IL-1β/IL-1R signaling during CCC. The cachexia that ensues from *T. cruzi* infection did not occur in *Il-1r* ***^−/−^*** mice, an unexpected finding, since it has been solely credited to TNF signaling (25). These findings follow the same pattern already found in *Toxoplasma gondii* infection, in which cachexia has been recently credited to IL-1β (41). Although blockade of IL-1R with anakinra for 30 days did not produce weight gain, it did result in improvement of clinical signals and reversed the increase in heart relative weight. These findings deserve further studies focusing on the role of IL-1β on cachexia and general health during *T. cruzi* infection to be confirmed.

A limitation of our work concerns the variation of the parasite burden after treatment of chronically infected mice with anakinra. An increase in parasite burden after IL-1β blockade could potentially compensate for the beneficial effects of such blockade on cardiac function. Although we cannot rule out variations in heart parasitism after treatment with anakinra, we do not believe it is the case since no detectable heart parasitism was found in HE sections from their hearts, but instead, we detected a decrease in the number of inflammatory cells infiltrating their hearts. Previous works showed lack of IL-1R expression does not prevent trypanocidal NO production by macrophages (18), but in our experiments, the absence of IL-1R expression produces greater parasitemia during the acute stage and we did not assess the chronic heart parasitism. There are currently no works systematically approaching the role of IL-1β in controlling established heart parasitism at the chronic stage of Chagas disease.

One could argue that treatment with anakinra is futile because once CCC is well-established, systolic dysfunction and arrhythmias cannot be reversed, since they result from the destruction of heart tissue. However, there are several demonstrations in the literature that cardiac dysfunction in experimental CCC can be reversed even when attempted late after infection. We have previously shown a great improvement in systolic function, an increase in heart rate, and partial reversal of right ventricle dilation and conduction blocks/arrhythmias after treating chronically infected mice with resveratrol (27). Treatment prorogation or treatment with resveratrol late after infection onset (120 dpi) also resulted in improvements in heart function. Treatment of chronically infected mice with Met-RANTES, an analog of CCL3 that blocks CCR1/CCR5 receptors, reduced PR and QTc prolongation, and partially reversed bradycardia (42). Our results here show that IL-1R blockade does not improve systolic function or reverse cardiac arrhythmias, indicating IL-1β/IL-1R signaling is not involved in its maintenance, as it is in other diseases (16, 17).

Strategies to reverse cardiac dysfunction at the chronic stage, such as IL-1R antagonists, are particularly suitable to treat Chagas disease since it is usually diagnosed late after infection. Currently, there are no specific treatments for CCC, and treatment is solely an extension of usual treatments for heart failure and arrhythmias. Despite shortening of QJ ventricle repolarization interval, a known substrate for arrhythmias, IL-1R blockade failed to reduce the incidence of arrhythmias/conduction blocks observed in EKG and the triggered activities upon extra-stimulation protocol (S_1_–S_2_) in CCC. Also, the improvement in EF in anakinra-treated mice was not accompanied by improvement in SV, thereby indicating failure to improve systolic dysfunction. Such failures argue nonexclusively for the following: (1) an irrelevant role for IL-1β in cardiac dysfunction; (2) an insufficient interval/IL-1β blockade to reverse established cardiac dysfunction; (and 3) a cooperative role for cytokines/factors in the maintenance of cardiac dysfunction. Since chronically infected *Il-1r* ***^−/−^*** mice presented an incidence of arrhythmias similar to infected WT on EKGs and a similar SV, we are left with hypotheses (1) and (3). Our data show that using anakinra alone will most likely not suffice as a therapeutical option to treat arrhythmias and heart failure in CCC and discourage any further attempts.

## Material and methods

### Ethics statement

This study was carried out in strict accordance with the recommendations of the Guide for the Care and Use of Laboratory Animals of the Brazilian National Council of Animal Experimentation (http://www.cobea.org.br/) and Federal Law 11.794 (08 October 2008; The Institutional Committee for Animal Ethics of UFRJ (CEUA, 005/2014)).

### Mice

Male and female C57BL/6 mice (6–8 weeks of age) obtained from the animal facilities of the National Center of Structural Biology and Bioimaging (CENABIO) of the Federal University of Rio de Janeiro, Brazil, were kept in a sterile environment under standard conditions (temperature and relative humidity of approximately 22°C ± 2°C and 58% ± 10%, respectively) and received food and water ad libitum. Mice were individually identified by ear tags.

### Mice infection and treatment

C57BL/6 WT and *Il-1r* ***^−/−^*** in the same genetic background were infected *via* i.p. with 50, 100, or 200 parasites of the Colombian strain of *T. cruzi*. Female (*n* = 23 WT; *n* = 15 *Il-1r* ***^−/−^***) and male (*n* = 4 WT; *n* = 3 *Il-1r* ***^−/−^***) mice were used in these experiments. In addition, the effect of blocking the IL-1 receptor was evaluated during the chronic phase of Chagas disease (240–270 dpi) in female mice with the administration of anakinra (Kineret, SOBI, Stockholm, Sweden), an IL-1R antagonist. In our prior experiments, we found intense cardiac effects using a 10-mg/kg dose in diabetes mellitus (17). Here, we first treated a group of mice with a 10-mg/kg dose. In the face of the lack of beneficial effect, we increased the dose to 25 mg/kg. Since the results were similar and there was no dose dependence, we plotted all these results together.

### Transthoracic echocardiography

To assess cardiac ventricular function, an echocardiogram was performed. Mice under deep isoflurane anesthesia (2% in oxygen) were trichotomized in the precordial region using depilatory cream. The study was performed using a 30-Mhz transducer with a Vevo 770 Ultrasound apparatus (Visual Sonics, Toronto, Canada). The left ventricle ejection fraction (LVEF) was calculated using Simpson’s method, chosen because of its fit with CD heart geometry and because it is commonly used to assess CD patients. The areas of the left and right ventricles during diastoles and systoles were obtained in B mode using a short-axis view at the level of the papillary muscles.

### Electrocardiography

EKG signals were recorded as previously described (27). Mice were sedated with diazepam (10 mg/kg), and electrodes were placed subcutaneously (DI derivation). Traces were recorded using the Power-Lab 2/20 System connected to an amplifier (Panlab Instruments, Spain) at a sample rate of 1 kHz.

The analysis of EKG recordings was performed in Trace-Watcher (43), a custom Lab-View–based Software (National Instruments, USA) designed and gently provided to us by Dr. Ariel Escobar (UC-Merced, USA). Briefly, EKG signals were filtered with a Gaussian filter at a 0.2-kHz cutoff value, then the baseline was corrected, and the signals were segmented in windows containing the whole cardiac cycle. The P wave, PR interval, and QJ duration were measured. Additionally, R peak locations were automatically detected and then adjusted by a trained observer using PhysioZoo software (44). Spontaneous cardiac arrhythmias were evaluated by a trained observer during the whole recording.

### Ventricular action potential recordings at a whole-heart level

To evaluate epicardial left ventricular action potentials (APs), sharp glass microelectrodes were used as previously described (16). Fifteen minutes prior to euthanasia, mice were injected with 1,000 UI of heparin (i.p., Cristalia, São Paulo, Brazil) to prevent clot formation. Animals were euthanized by cervical dislocation. Hearts were then quickly removed and gently washed in Tyrode’s solution containing (in mM) the following: 140 NaCl, 5.4 KCl, 2.0 CaCl_2_, 1.0 MgCl_2_, 10.0 d-glucose, 0.33 Na_2_HPO_4_, 10.0 HEPES (pH 7.4 ± 0.02 adjusted NaOH at 37.0°C). The aorta was cannulated in a Langendorff system and immediately perfused with oxygenated Tyrode’s solution. After 10–15 min of the stabilization period, the temperature was progressively increased from room temperature to 35.5°C–37°C, and blebbistatin 4 µM (Selleckchem, USA) was added to Tyrode’s solution to avoid mechanical artifacts during the AP recording. AP was recorded using a sharp borosilicate microelectrode (WPI, USA) filled with KCl at 3M and an approximate resistance of 15–20 MΩ connected to a high impedance microelectrode amplifier (Electro 705, WPI, USA). The signal was digitalized at a 2.5-kHz sampling frequency and recorded using LabView custom-designed software. All APs were recorded by the S_1_S_1_ pacing protocol at 5 Hz by a pulse generator (Digitimer DS2A, UK).

APs were analyzed in a custom LabView-based program (Trace-Watcher) developed and kindly provided by Dr. Ariel Escobar (43). First, signals were filtered with a Gaussian filter at 2 kHz. APs were individualized and normalized between 0 and 1. APD was measured at 30%, 50%, 70%, and 90% of repolarization. The average of approximately 180 AP traces was considered representative for each animal.

To test ventricular susceptibility to arrhythmias, we performed an extra-stimulation protocol. Hearts were paced at 5 Hz (S_1_) and then an extra stimulus (S_2_) was added. The S_1_–S_2_ period was gradually reduced. When an arrhythmic event was evoked, the stimulus was torn off.

### Statistics

Statistical analysis was performed using Prism v 8.0 (GraphPad, USA). Data are shown in bar graphs as mean and standard error of the mean (SEM). Means were compared using Student’s *t*-test. The comparison between proportions of arrhythmic events across groups was calculated using Fisher’s exact test. *p*-values ≤0.05 were considered statistically significant. Significant differences are marked in each graph (^*^).

## Data availability statement

The datasets analyzed for this study are available from the corresponding authors upon reasonable request.

## Ethics statement

This study was carried out in strict accordance with the recommendations of the Guide for the Care and Use of Laboratory Animals of the Brazilian National Council of Animal Experimentation (http://www.cobea.org.br/) and Federal Law 11.794 (October 8, 2008); The institutional Committee for Animal Ethics of UFRJ (CEUA, 005/2014).

## Author contributions

CP and EM designed the study. CO, OM-L, D-FV, IPR, and HM-S performed the experiments. CO, OM-L, MB, CP, and EM analyzed the data. OM-L, CP and EM wrote the first version of the manuscript. All authors reviewed critically the manuscript and approved its final version of the manuscript.

## Funding

OM-L received doctoral scholarship from Carlos Chagas Filho Foundation for Supporting Research in the State of Rio de Janeiro (FAPERJ) E26/200.396/2020. MB and EM received personal PQ CNPq grants. EM received grants E-26/210.155/2020, E-26/203.169/2017, E-26/210.191/2020, and E-26/210.253/2020; CNPq 310681/2018-9. MB received financial support from CNE E-26/201.128/2022(272688) and E-26/211.564/2019 (252360) e Pensa Rio FAPERJ.

## Conflict of interest

The authors declare that the research was conducted in the absence of any commercial or financial relationships that could be construed as a potential conflict of interest.

## Publisher’s note

All claims expressed in this article are solely those of the authors and do not necessarily represent those of their affiliated organizations, or those of the publisher, the editors and the reviewers. Any product that may be evaluated in this article, or claim that may be made by its manufacturer, is not guaranteed or endorsed by the publisher.

## Supplementary material

Supplementary Figure 1 Heart tissue inflammatory infiltrates and collagen. Wild-type (WT) (C57BL/6 background) were infected with Colombian strain of *T. cruzi* (100 parasites) from 270 dpi and treated with an IL-1R antagonist, anakinra (10mg/Kg, i.p.), daily for 30 days. **(A)** Inflammatory infiltrates per microscope field (400x magnification) obtained from Hematoxylin-Eosin stained sections. Twenty sections were analyzed per mouse and data represent mean ± SEM from 9-10 mice per group. **(B)** Representative pictures from Trichrome Masson-stained sections. Sections from 9-10 mice were analyzed per group.
