# Supplementary material for: IL-1β is not critical to chronic heart dysfunction in mice with Chagas disease
Source: Front Immunol. 2022 Oct 14;13:1010257. doi: 10.3389/fimmu.2022.1010257 (PMC9627615; doi:10.3389/fimmu.2022.1010257)
Supplement: Supplementary Figure 1 — Heart tissue inflammatory infiltrates and collagen. Wild-type (WT) (C57BL/6 background) were infected with Colombian strain of T. cruzi (100 parasites) from 270 dpi and treated with an IL-1R antagonist, anakinra (10mg/Kg, i.p.), daily for 30 days. (A) Inflammatory infiltrates per microscope field (400x magnification) obtained from Hematoxylin-Eosin stained sections. Twenty sections were analyzed per mouse and data represent mean ± SEM from 9-10 mice per group. (B) Representative pictures from Trichrome Masson-stained sections. Sections from 9-10 mice were analyzed per group. [file DataSheet_1.pdf]

Arrhythmias	WT (n = 14)		WT + Tc (n = 22)		IL-1R (n = 9)		IL-1R + Tc (n = 10)		Tc + AK (n = 12)	
	N	%	N	%	N	%	N	%	N	%
**Atrioventricular block**	0	0.0	14	63.6	0	0.0	7	70.0	9	75.0
**First degree**	0	0.0	7	31.8	0	0.0	2	20.0	5	41.7
**Second degree**	0	0.0	3	13.6	0	0.0	3	30.0	0	0.0
**Third degree**	0	0.0	4	18.2	0	0.0	2	20.0	4	33.3
**Tachyarrhythmias**	1	7.1	12	54.5	1	11.1	6	60.0	2	16.6
**Supraventricular extrasystole**	0	0.0	3	13.6	1	11.1	1	10.0	0	0.0
**Ventricular extrasystole**	1	7.1	2	9.1	0	0.0	2	20.0	0	0.0
**Couplet**	0	0.0	3	13.6	0	0.0	0	0.0	1	8.3
**Triplet**	0	0.0	2	9.1	0	0.0	3	30.0	1	8.3
**Bigeminy**	0	0.0	2	9.1	0	0.0	0	0.0	0	0.0
**Supraventricular tachycardia**	0	0.0	0	0.0	0	0.0	1	10.0	1	8.3
**Junctional Rythm**	0	0.0	0	0.0	0	0.0	1	10.0	0	0.0
**Atrial Fibrillation**	0	0.0	0	0.0	0	0.0	1	10.0	0	0.0
**Ventricular tachycardia**	0	0.0	3	13.6	0	0.0	1	10.0	1	8.3
**Total**	1	7.1	19	86.4	1	11.1	8	80.0	10	83.3

A.

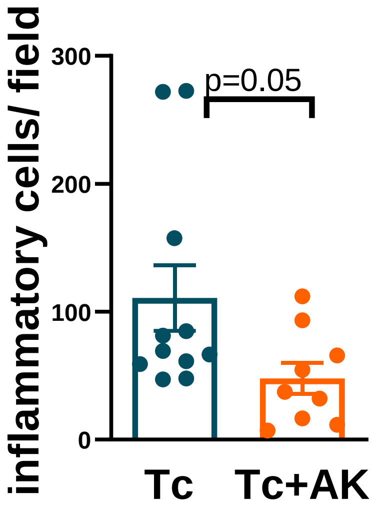

inflammatory cells/ field
300
200
100
0
p=0.05
Tc
Tc+AK

B.

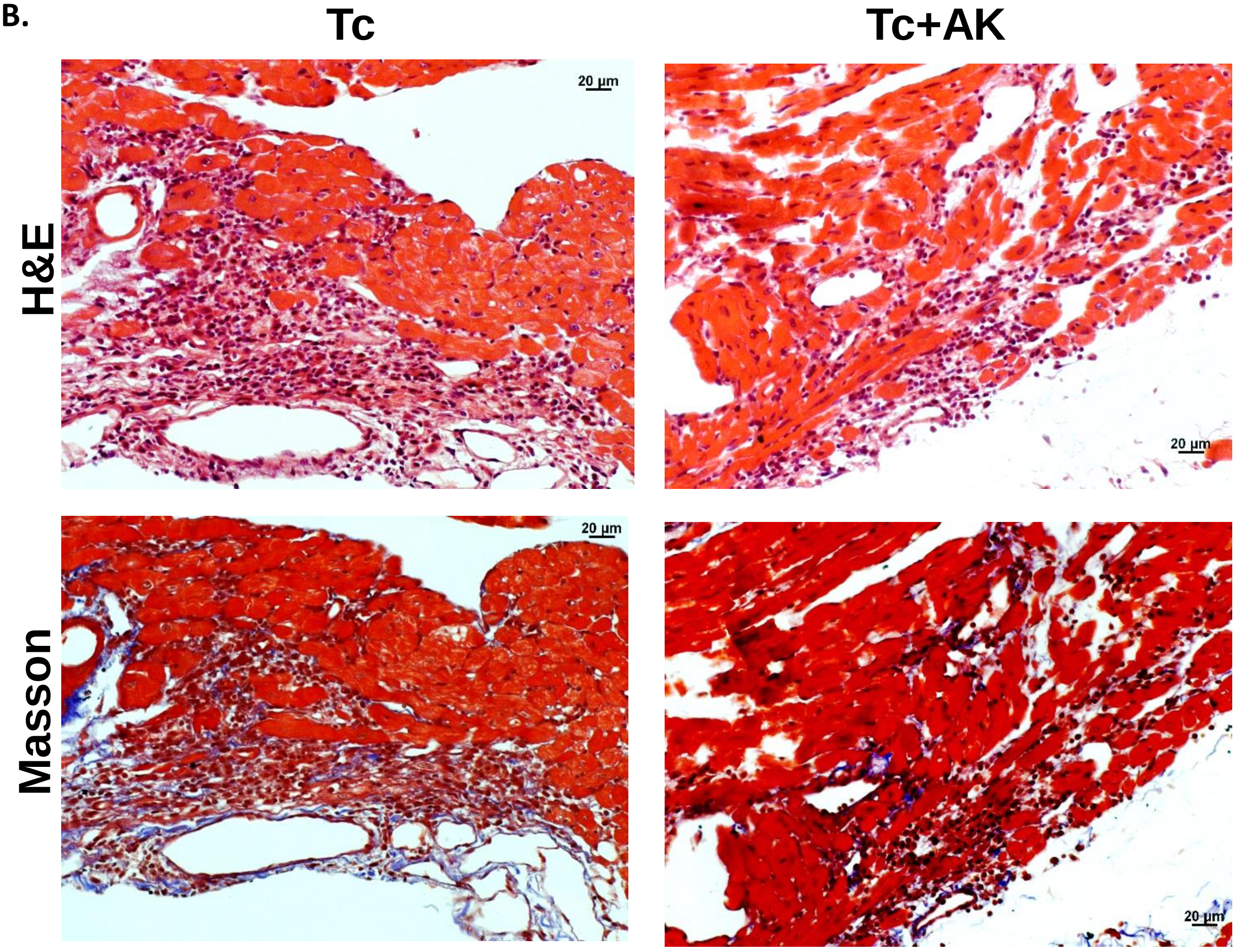

Tc
Tc+AK
H&E
Masson
20 µm
20 µm
20 µm
20 µm
